# Efficacy of epidermal growth factor receptor–tyrosine kinase inhibitors for lung squamous carcinomas harboring EGFR mutation: A multicenter study and pooled analysis of published reports

**DOI:** 10.18632/oncotarget.17915

**Published:** 2017-05-17

**Authors:** Yongmei Liu, Yan Zhang, Li Zhang, Bin Liu, Yongsheng Wang, Xiaojuan Zhou, Yanying Li, Qian Zhao, Youling Gong, Lin Zhou, Jiang Zhu, Zhenyu Ding, Jin Wang, Feng Peng, Meijuan Huang, Lu Li, Li Ren, You Lu

**Affiliations:** ^1^ Department of Thoracic Oncology, Cancer Center and State Key Laboratory of Biotherapy, West China Hospital, Sichuan University, Chengdu, China; ^2^ Department of Medical Oncology, Sun Yat-Sen University Cancer Center, Guangzhou, China; ^3^ Pulmonary Tumor Ward, Sichuan Cancer Hospital, Chengdu, China; ^4^ West China School of Medicine, Sichuan University, Chengdu, China

**Keywords:** squamous cell carcinoma, EGFR mutation, epidermal growth factor receptor-tyrosine kinase inhibitor

## Abstract

Epidermal growth factor receptor (EGFR) mutations are common in lung adenocarcinoma (ADC) but rare in squamous cell carcinoma (SQC). The efficacy of EGFR-tyrosine kinase inhibitors (EGFR-TKIs) for SQC with EGFR mutations is unclear. The aim of this study was to evaluate the efficacy of EGFR-TKIs for these patients. We performed a retrospective matched-pair case-control study from 3 cancer centers, including 44 SQC and 44 ADC patients with EGFR mutation who were treated with EGFR-TKI. Subsequently, we performed a pooled analysis on the efficacy of EGFR-TKIs for EGFR-mutant SQC in 115 patients, including 71 patients selected from 25 published reports. In our multicenter study, EGFR-mutant SQC and ADC patients had similar objective response rate (ORR) (43.2% vs. 54.5%, *p* = 0.290), but SQC patients had lower disease control rate (DCR) (71.3% vs. 100%, *p* = 0.001), significant shorter median progression free survival (PFS) (5.1 vs. 13.0 months, *p* = 0.000) and median overall survival (OS) (17.2 vs. 23.6 months, *p* = 0.027). In pooled analysis, the ORR, DCR, PFS and OS of SQC patients were 39.1%, 71.3%, 5.6 months and 15.0 months, respectively. Performance status was the only independent predictor of PFS and erlotinib treatment was associated with a better survival. In conclusion, EGFR-TKI was less effective in EGFR-mutant SQC than in ADC but still has clinical benefit for SQC patients. Further study is need to evaluate the using of EGFR-TKIs in these SQC patients.

## INTRODUCTION

Epidermal growth factor receptor-tyrosine kinase inhibitors (EGFR-TKIs) are currently recommended as the standard first line treatment for non-small cell lung cancer (NSCLC) harboring EGFR-sensitive mutations. The majority of clinical data regarding EGFR-TKIs in EGFR-mutant population are derived from lung adenocarcinoma (ADC).

The percentage of EGFR mutations in lung squamous cell carcinoma (SQC) is only about 10% in persons of Asian descent [1] and < 5% in western countries [2]. Our literature review only identified small case series studies and case reports about the sensitivity of EGFR-TKI in SQC with EGFR mutation in the literature. The objective response rates (ORR), disease control rates (DCR) and median progression free survival rates (PFS) in these studies were 25.0–26.7%, 66.7–71.0% and 1.4–6.3 months, respectively [3–5]. EGFR-TKI seems to be less effective in SQC than in ADC and the role of EGFR-TKI in EGFR-mutant SQC remains to be debated.

We therefore performed a matched-pair case-control study retrospectively to investigate the different efficacy of EGFR-TKI in EGFR-mutant SQC and ADC patients from 3 cancer centers. Subsequently, a pooled analysis that included our own data and data obtained from previous studies at other institutions was carried out to evaluate the role of EGFR-TKIs in EGFR-mutant SQC, which represents the largest study to our knowledge of this patient cohort.

## RESULTS

### Patient characteristics

The initial matched-pair case-control study included 88 NSCLC patients from 3 cancer centers (44 SQC and 44 ADC). Among them, 52 patients were male, 46 patients were non-smokers and 62 patients had an Eastern Cooperative Oncology Group performance status (ECOG PS) of 0–1. The median age was 56 years (range: 32–80). Patient characteristics are shown in Table 1. Each group included 28 patients with a 19-DEL mutation, 15 with a L858R mutation and 1 with a G719X and S768I mutation simultaneously; 19 patients received EGFR-TKIs as first line treatment and 25 patients had prior chemotherapy; 26 patients received gefitinib, 14 received erlotinib, and 4 received icotinib.

**Table 1:** Baseline characteristics

	SQC		ADC	
	*n* = 44	Percent (%)	*n* = 44	Percent (%)
Gender				
Male	26	59.1	26	59.1
Female	18	40.9	18	40.9
Age(years)				
≥ 70	38	86.4	38	86.4
< 70	6	13.6	6	13.6
ECOG PS				
< 2	31	68.9	31	68.9
≥ 2	14	31.1	14	31.1
Smoking status				
Smoker(current/former)	23	52.3	23	52.3
Non-smoker(never)	21	47.7	21	47.7
EGFR mutation type				
19-DEL	28	63.6	28	63.6
L858R	15	34.1	15	34.1
719X/S768I	1	2.3	1	2.3
Prior chemotherapy				
0	19	43.2	19	43.2
≥ 1	25	56.8	25	56.8
EGFR-TKI treatment				
Gefitinib	26	59.1	26	59.1
Erlotinib	14	31.8	14	31.8
Icotinib	4	9.1	4	9.1

SQC, squamous cell carcinoma; ADC, adenocarcinoma; ECOG PS, Eastern Cooperative Oncology Group performance status; EGFR, epidermal growth factor receptor; TKI, tyrosine kinase inhibitor.

The subsequent pooled analysis included 115 EGFR-mutant lung SQC patients treated with EGFR-TKIs. Seventy-one patients were selected from 25 reports [3, 6–29]; 55 patients (71.8%) in 19 of these reports were from East Asian countries. Definitive data of age, gender, smoking history, PS, status of EGFR mutation and drugs of EGFR-TKIs treatment could be extracted in 38(53.5%), 56(78.9%), 51(71.8%), 15(21.1%), 59(83.1%) and 42(59.2%) of the 71 patients, respectively. Patient characteristics for this cohort are summarized in Table 2. The median age was 61.5 years. Gender, smoking history, and PS were: male (63/100, 63.0%), female (37/100, 37.0%); never smoker (44/95, 46.3%), smoker (51/95, 53.7%); PS < 2 (42/59, 71.2%), PS ≥ 2 (17/59, 28.8%). There were 55 patients (55/103, 53.4%) with 19-DEL mutations, 31 patients (31/103, 30.1%) with L858R mutations and 17 patients (17/103, 16.5%) with other mutations. Fifty-eight (58/86, 67.4%) patients received gefitinib, 24 patients (24/86, 27.9%) received erlotinib and 4 patients (4/86, 4.7%) received icotinib. Twenty-seven patients (27/82, 32.9%) were treated with TKIs as first line treatment and 55 patients (55/82, 67.1%) had prior chemotherapy.

**Table 2:** Characteristics of the squamous cell carcinoma patients included in the pooled analysis (*n* = 115)

	*n*	%
Gender	100	
Male	63	63.0
Female	36	37.0
Age(years)	82	
Median	61.5	
Range	29-82	
ECOG PS	59	
< 2	42	71.2
≥ 2	17	28.8
Smoking status	95	
Smoker(current/former)	51	53.7
Non-smoker(never)	44	46.3
EGFR mutation type	103	
19-DEL	55	53.4
L858R	31	30.1
Other mutations	17	16.5
Prior chemotherapy	82	
0	27	32.9
≥ 1	55	67.1
EGFR-TKI treatment	86	
Gefitinib	58	67.4
Erlotinib	24	27.9
Icotinib	4	4.7
Efficacy of EGFR-TKIs	115	
CR/PR	45	39.1
SD	37	32.2
PD	33	28.7

ECOG PS, Eastern Cooperative Oncology Group performance status; EGFR, epidermal growth factor receptor; TKI, tyrosine kinase inhibitor.

### Response and survival

**Figure 1:**
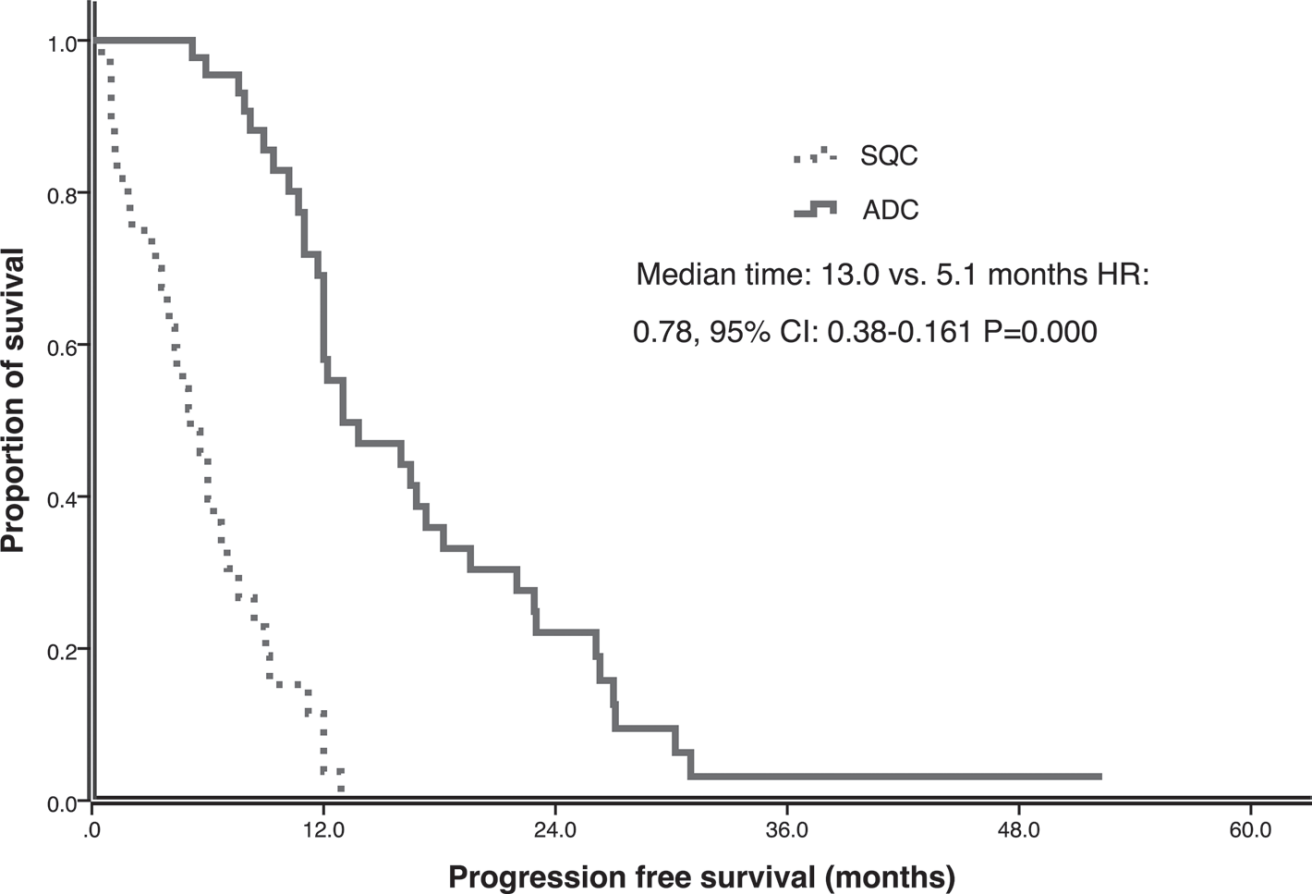
Progression free survival (PFS) of ADC and SQC in multicenter study

**Figure 2:**
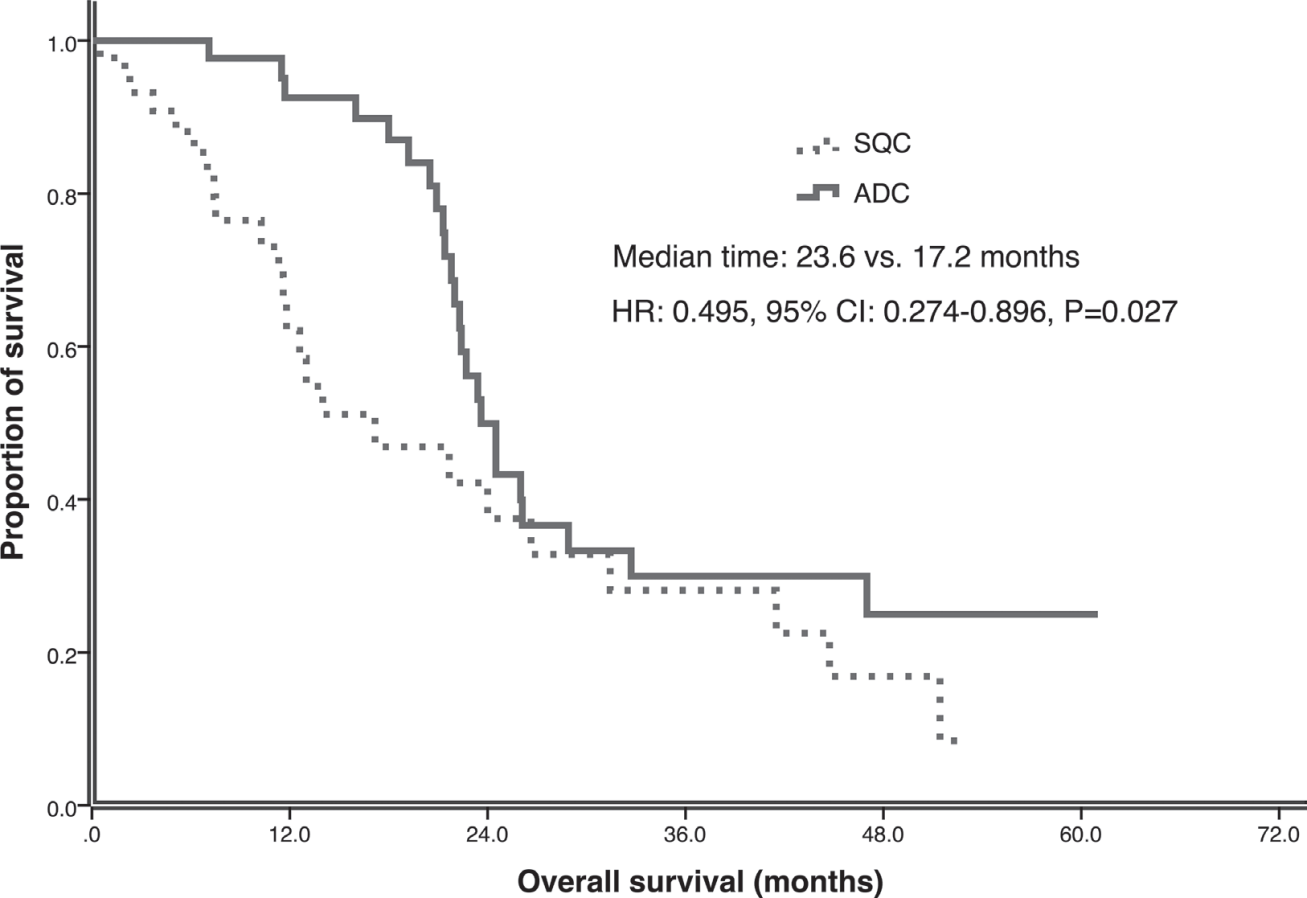
Overall survival (OS) of ADC and SQC in multicenter study

In the matched-pair case-control study, the median follow-up time for all the 88 patients was 41.1 months (range, 0.5–61.0 months) upto the study closing date of December 30, 2015. For SQC patients 19 had partial responses (PR), 15 had stable disease (SD) and 10 had progressive disease (PD) after EGFR-TKI treatment, which resulted in ORR of 43.2% and DCR 77.3%. For ADC patients, ORR was 54.5% and DCR was 100%, including 24 PR and 20 SD. There was no significant difference of ORR between SQC and ADC groups (43.2% versus 54.5%, *P* = 0.290), but SQC group had lower DCR than ADC group (77.3% vs. 100%, *P* = 0.001). By the time of the final analysis, 9 patients had not experienced progression and 20 patients were still alive in both SQC and ADC group. The median PFS of all the patients was 10.7 months (95% CI: 8.38–13.03). SQC group had significant shorter PFS than ADC group (5.1 vs. 13.0 months, *p* = 0.000) (Figure 1). The median OS of all the patients was 22.7 months (95% CI: 20.53- 24.87). The OS of SQC group and ADC group had statistical significant difference (17.2 vs. 23.6 months, *p* = 0.027) (Figure 2).

In the pooled analysis, tumor response was identified in 71 patients from the published reports. The ORR and DCR were 39.1% and 71.3%, respectively, of all the 115 SQC patients. PFS was identified in 28 patients from the published reports. The PFS analysis was performed in 72 SQC patients. The median PFS of all the patients was 5.6 months (95% CI: 3.93–7.27 months). Better PS (ECOG 0–1) was associated with better PFS both in univariate and multivariate analysis (Table 3). OS was extracted in 26 patients from the reports. The OS analysis was performed in 70 SQC patients. The median OS of all the patients was 15.0 months (95% CI: 8.15–21.85 months). The patients treated with erlotinib had longer OS than those treated with gefitinib both in univariate and multivariate analysis (Table 4).

**Table 3:** Association between clinical factors and the PFS

	PFS (months)	Univariate analysis, P^a^	Multivariate analysis, P^b^
Gender		0.327	
Male	6.3		
Female	5.0		
Smoking status		0.498	
Smoker(current/former)	6.3		
Non-smoker(never)	5.1		
EGFR mutation type		0.217	
19-DEL	5.0		
L858R	7.0		
ECOG PS		0.000	0.001 (HR:4.141, 95%CI: 1.854-9.248)
< 2	6.7		
≥ 2	3.1		
Prior chemotherapy		0.820	
0	6.0		
≥ 1	5.0		
EGFR-TKI treatment		0.116	
Gefitinib	4.4		
Erlotinib	7.0		

ECOG PS, Eastern Cooperative Oncology Group performance status; EGFR, epidermal growth factor receptor; TKI, tyrosine kinase inhibitor; PFS, median progression free survival; ^a^Log-rank test. ^b^Cox regression test

**Table 4:** Association between clinical factors and the OS

	OS (months)	Univariate analysis, P^a^	Multivariate analysis, P^b^
Gender		0.667	
Male	17.2		
Female	15.0		
Smoking status		0.481	
Smoker(current/former)	17.2		
Non-smoker(never)	14.0		
EGFR mutation type		0.905	
19-DEL	14.0		
L858R	24.0		
ECOG PS		0.067	
< 2	17.2		
≥ 2	6.2		
Prior chemotherapy		0.363	
0	12.6		
≥ 1	19.3		
EGFR-TKI treatment		0.047	0.025 (HR: 0.391, 95%CI: 0.172-0.887)
Erlotinib	24.0		
Gefitinib	11.6		

ECOG PS, Eastern Cooperative Oncology Group performance status; EGFR, epidermal growth factor receptor; TKI, tyrosine kinase inhibitor; OS, median overall survival; ^a^Log-rank test. ^b^Cox regression test.

## DISCUSSION

In this study, we investigated the efficacy of EGFR-TKI treatment in lung SQC harboring EGFR mutation. We found that the ORR, DCR, PFS and OS of EGFR-mutated lung SQC treated with EGFR-TKI were 39.1%, 71.3%, 5.6 months and 15.0 months, respectively.

In our multicenter matched-pair case-control study, SQC patients had significant lower DCR, shorter PFS and OS than ADC patients. Although the mechanism behind the efficacy difference between ADC and SQC is unclear, there is now evidence from previous studies, including the TCGA study, revealing a number of genomic differences between ADC and SQC [30]. Gene amplifications of 3q including SOX2, TP63, PIK3CA, and EPHB3 were observed in as many as 86% of SQC samples but only 21% of adenocarcinoma samples [31]. Allelic losses [32] or hypermethylations [33] on the 3p region has been observed more frequently in tumors with a squamous histology compared with adenocarcinoma, involving some tumor suppressor genes such as RASSF1A, FUS1, VHL, and FHIT. Meanwhile activations of other signaling pathways, such as ERBB2 [34] and PIK3CA [35, 36] may mediate resistance of lung cancers to EGFR-targeting therapies. We theorize that multiple signaling pathways are activated in the development and progression of lung SQC, while ADC may depend on a key pathway, such as EGFR, ALK or ROS-1. Accordingly, the efficacy is inferior in SQC patients when therapy to block a single pathway is used. However, we feel that the utility of EGFR-TKI in SQC should be studied further.

We found that SQC patients treated with EGFR-TKI had the similar ORR to ADC patients. Approximately one third of the EGFR-mutant SQC patients obtained a PFS longer than 6 months and an OS longer than 1 year which may be due to the efficacy of EGFR-TKIs. Some EGFR-mutant SQC could benefit from EGFR-TKI treatment and EGFR-TKI is an important intervention for these patients. Further study is needed to identify these patients that may benefit.

The incidence of EGFR mutations in lung SQC may differ in Asian and non-Asian populations. Previous reports revealed that the incidence of EGFR mutations in western populations is low, ranging from 0% to 3.6% [37, 38]. However, the frequency of EGFR mutation is much higher in Asian populations [1, 3, 5]. The incidence of SQC harboring sensitive EGFR mutation was 13.3% (63/249) in Japan [3] and 17.8% (109/614) in another study from Chin a [5]. In our center, the EGFR-mutant incidence of lung SQC was 6.1% (22/359). Therefore, we consider routine EGFR mutation testing for all Asian lung SQC patients.

This study is a retrospective multiple center and comprehensive review of the published literature. The validation of the results relies on higher level evidence, such as randomized controlled trials. Although this study includes all available data we identified in the literature, the sample size is still insufficient. Large sample studies would help generate more robust conclusions.

In conclusion, EGFR-TKI was less effective in EGFR-mutant SQC than in ADC but still has clinical benefit for some SQC patients. We present the largest pooled analysis to identify the clinical profiles of EGFR-TKI application for EGFR-mutant SQC, which may provide valuable information for treatment selection.

## MATERIALS AND METHODS

### Patients

From January 2004 to October 2015, 44 EGFR-mutant SQC patients treated with EGFR-TKI (gefitinib 250 mg/day, erlotinib 150mg/day or icotinib 125 mg tid) were selected from 3 cancer centers including the cancer centers of West China Hospital, Sichuan University, Sun Yat-sen University Cancer Center and Cancer Hospital of Sichuan Province, China. The patients were diagnosed by bronchofiberscope or percutaneous lung biopsy. The pathological diagnosis was confirmed by light microscopy and immunohistochemistry (IHC) and verified by stains for P40, thyroid transcription factor (TTF)-1 and Napsin A (negative, positive, and negative, respectively). EGFR mutations were identified by real-time Amplification Refractory Mutation System (ARMS) quantitative PCR analysis using AmoyDxTM EGFR Mutation Detection Kit (Amoy Diagnostics Co., LTD, China) and EGFR Scorpion ARMS Kit (DxS Ltd, Manchester, UK). All patients have the confirmed status of EGFR mutation.

EGFR-mutant ADC patients were pair matched to selected SQC patients from our lung cancer database according to seven variables: gender (male, female); age (< 70 years, ≥ 70 years); smoking history (non-smokers, current or former smokers); ECOG PS (< 2, ≥ 2); status of EGFR mutation (19-DEL, L858R, G719X/S768I); drugs of EGFR-TKI (gefitinib, erlotinib, icotinib) and prior chemotherapy (0, ≥ 1). Clinical data of each patients was well recorded.

Tumor response was determined by the Response Evaluation Criteria in Solid Tumors (RECIST) version 1.1, including complete response (CR), partial response (PR), stable disease (SD), and progressive disease (PD). The treatment response was evaluated 1 month after the initiation of EGFR-TKI therapy and then every 2 months. This retrospective analysis was approved by the Institutional Review Board of the above stated hospitals and signed consent was obtained from each patient for the use of tissue in molecular analysis.

In order to further investigate the efficacy of EGFR-TKI for EGFR-mutant lung SQC, we performed a systematic search of the PUBMED database to identify all clinical trials and case reports that contained EGFR-mutant SQC patients treated with EGFR-TKI. The search strategy included articles from April 2004 to November 2015 indexed under the subject headings EGFR, mutation, and lung cancer. We did not restrict the search on the type of publication or periodical. All published reports that described the efficacy of EGFR-TKIs for EGFR-mutant SQC were selected. From the author's publications, we extracted the data of the SQC patients. If the data important for us to analyze was not shown in the article, we wrote to the authors asking for help to obtain the data. 71 patients were identified by the above strategy.

### Statistical analysis

PFS was calculated from the date of initiation of EGFR-TKIs to the time of disease progression. Overall survival (OS) was calculated from the time of beginning receiving EGFR-TKIs until the date of death. Survival curves were estimated by the Kaplan–Meier method and compared by the log–rank test. Multivariate analysis was performed using Cox regression test. Comparison of the qualitative data was done by Pearson χ^2^ or the Fisher exact test. *P* values < 0.05 was considered statistically significant. All statistical analyses were performed with SPSS 22.0.
